# Clinical value of combined plasma brain natriuretic peptide and serum cystatin C measurement on the prediction of heart failure in patients after acute myocardial infarction

**DOI:** 10.1590/1414-431X2023e12910

**Published:** 2023-09-22

**Authors:** Rui Liu, Changzheng Gao

**Affiliations:** 1 Department of Cardiology, The Affiliated Hospital of Jiangnan University, Wuxi, Jiangsu, China

**Keywords:** Heart failure, Percutaneous coronary intervention, Acute myocardial infarction, Cystatin C, Brain natriuretic peptide

## Abstract

This research investigated the predictive value of combined detection of brain natriuretic peptide (BNP) and cystatin C (Cys C) in heart failure after percutaneous coronary intervention (PCI) in patients with acute myocardial infarction (AMI). Sixty-five AMI patients complicated by heart failure (HF) after PCI and 79 non-heart failure (non-HF) patients were involved in this research. The levels of Cys C and BNP were measured. Risk factors for heart failure in AMI patients after PCI were analyzed by multivariate logistic regression analysis. Efficacy of BNP and Cys C on predicting heart failure were analyzed by receiver operating characteristic (ROC) curve. Cys C and BNP levels were significantly higher in the HF group than in the non-HF group. BNP and Cys C levels were the independent influencing factors causing heart failure within one year after PCI. The area under the predicted curve (AUC) of Cys C, BNP, and combined Cys C and BNP were 0.763, 0.829, and 0.893, respectively. The combined detection of Cys C and BNP was highly valuable in predicting heart failure in AMI patients after PCI, which can be regarded as the serum markers for diagnosis and treatment of heart failure.

## Introduction

Acute myocardial infarction (AMI) refers to hypoxia-ischemia-induced myocardial injury and necrosis caused by sudden reduction or interruption of blood flow due to the blockage of coronary arteries (1). AMI can cause chest pain, arrhythmias, and other complications with rapid onset and disease progression, which can be life-threatening if not intervened in time (2).

The widespread use of percutaneous coronary intervention (PCI) has significantly reduced mortality in patients with AMI, but the development of heart failure after the procedure severely affects the recovery of patients after treatment (3). Early recognition of the onset of heart failure after AMI and intervention within the appropriate time window is key to improving patient prognosis (4).

Brain natriuretic peptide (BNP) is the current hematological gold standard for acute heart failure diagnosis (5). BNP is synthesized and secreted in large amounts in response to myocardial ischemia, ventricular volume loading, and increased ventricular wall tension. It correlates with heart failure severity and is an objective indicator for heart failure diagnosis (6). However, whether BNP concentrations after AMI have correlation with heart failure is controversial.

Most of the heart failure patients have inadequate renal blood flow (7). Serum cystatin C (Cys C) measurement is a recently developed parameter for evaluating renal function and is more accurate and sensitive to early minor glomerular filtration rate changes than blood creatinine levels (8). Therefore, this study explored the value of BNP and Cys C concentrations in AMI patients undergoing PCI in predicting heart failure.

## Material and Methods

### Patients

In this research, a total of 153 AMI patients admitted to the Affiliated Hospital of Jiangnan University were recruited. Patients with AMI received standard loading dose medications (300 mg aspirin and 180 mg ticagrelor or 300 mg clopidogrel) and were treated with PCI. PCI procedures were performed by experienced cardiologists.

Acute heart failure in AMI patients was diagnosed by clinical manifestations and physical examination: 1) typical clinical manifestations of new-onset heart failure (such as fatigue and varying degrees of dyspnea); 2) accelerated heart rate or the presence of gallop rhythm, and auscultation of the chest revealed moist rales; 3) chest X-ray showing pulmonary congestion, pulmonary edema, and pleural effusion; or 4) cardiogenic shock.

Inclusion criteria were age ≥8 years, time to reperfusion (onset to balloon dilation) <12 h, patients treated with PCI, and patients that had segment elevation (ST) or non-ST segment elevation AMI by significant coronary artery stenosis. Exclusion criteria were patients that had combined old myocardial infarction or chronic heart failure, combined pulmonary heart disease or pulmonary hypertension, severe hepatic or renal insufficiency or cranial diseases, or estimated life expectancy <1 year.

**Figure 1:**
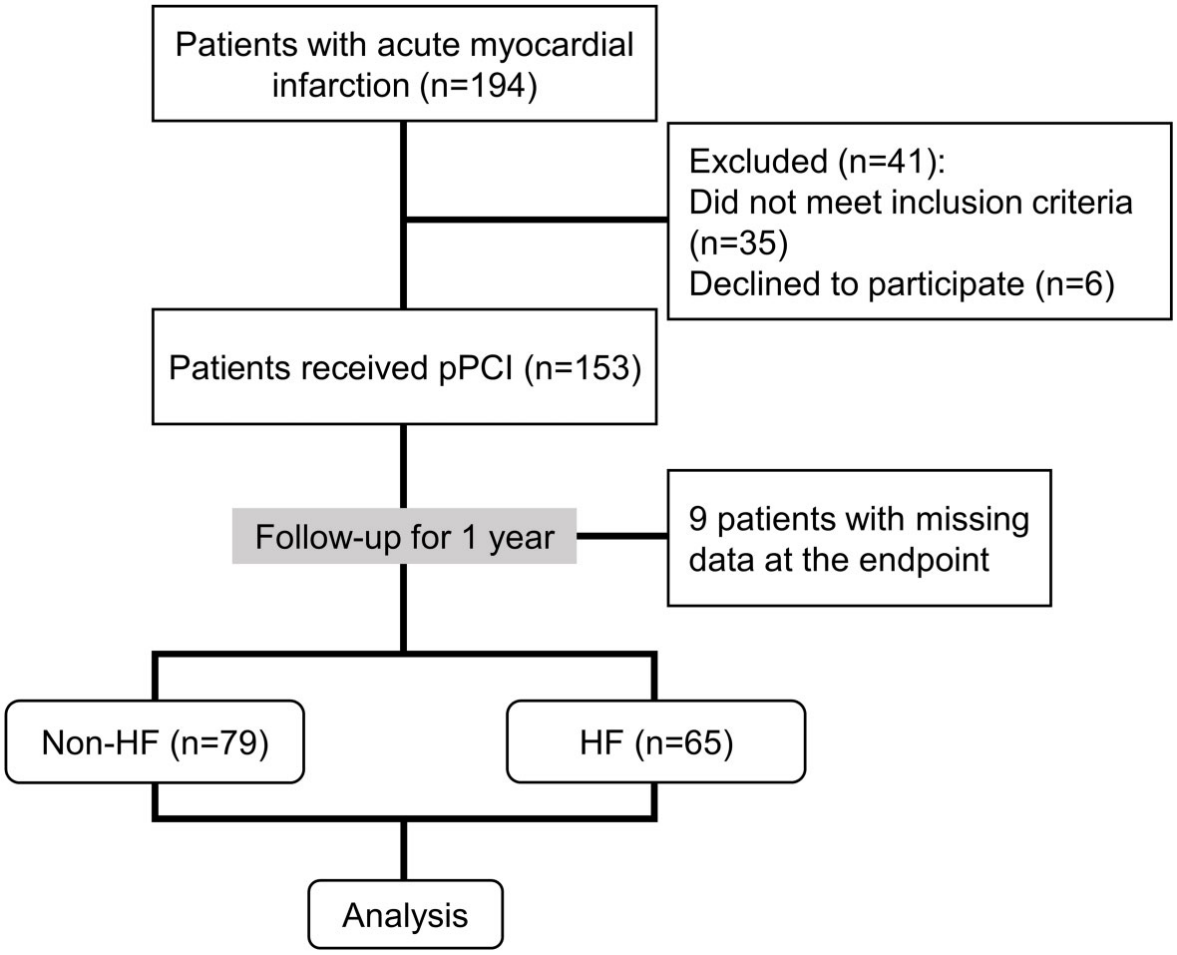
Study design. PCI: percutaneous coronary intervention; HF: heart failure.

Figure 1 shows the study flowchart. Of 194 patients with AMI, 153 were recruited for this research. Thirty-five patients were excluded and 6 patients declined to participant. After a one-year follow-up, 9 patients were lost. Blood sample collection, clinical examination, and echocardiography were performed. Of the 144 patients, 65 had heart failure (HF group) and 79 had no heart failure (non-HF group). The endpoint of our study was new heart failure onset at one-year follow-up. New-onset heart failure was defined as heart failure-related hospitalization or ambulatory diagnosis of heart failure. All the protocols were approved by the Affiliated Hospital of Jiangnan University, and informed consent forms were obtained from all patients.

### Biochemical testing

Blood samples were collected from patients at 48 h after PCI. All the laboratory tests were performed by the emergency laboratory of our hospital. BNP was evaluated by Elecsys electrochemiluminescent immunoassay (Roche, Switzerland). Cys C was measured using the Human Cystatin C ELISA Kit (Multi Sciences, China).

### Echocardiography

The left ventricular ejection fraction (LVEF), left ventricular end-diastolic volume (LVEDV), and left ventricular end-diastolic diameter (LVEDD) of patients were detected by echocardiography.

### Statistical analysis

The data are reported as means±SD or n (percentage). The differences between groups were compared by Mann-Whitney test or Fisher's exact test. Multivariate logistic analysis was used to identify independent predictors of heart failure during a 1-year period, with heart failure occurrence (0=no, 1=yes) as the dependent variable and age, BMI, gender, diabetes, hypertension, hyperlipidemia, chronic obstructive pulmonary disease (COPD), pre-stroke, chronic kidney disease, heart rate, blood pressure, echocardiographic parameters, total cholesterol (TC), triglycerides (TG), high-sensitivity C-reactive protein (hs-CRP,) BNP, creatine kinase-muscle/brain (CK-MB), and Cys C as independent variables. The stepwise method was used to exclude irrelevant items (P>0.05). Receiver operating characteristic (ROC) curve was used to evaluate the prediction efficacy of serum BNP and Cyc C levels in heart failure. P<0.05 was considered statistically significant. Data analysis in this study was conducted using the SPSS 23.0 software (IBM, USA).

## Results

### Demographic and clinical characteristics of patients

Demographic and clinical characteristics of patients are shown in Table 1. Patients in the HF group were older than in the non-HF group (67.4±8.1 *vs* 59.7±7.8 years, P=0.039). Body mass index (BMI), gender, diabetes, hypertension, hyperlipidemia, prior stroke, chronic kidney disease, and COPD had no significant difference between the two groups (all P>0.05).

**Table 1:** Demographic and clinical characteristics of patients with and without new heart failure (HF) onset at one-year follow-up after acute myocardial infarction (AMI).

	Non-HF (n=79)	HF (n=65)	P value
Age (years)	59.7±7.8	67.4±8.1	0.039
Body mass index (kg/m^2^)	23.8±3.3	24.6±2.9	0.198
Male	48 (60.8%)	31 (47.7%)	0.132
Female	31 (39.2%)	34 (52.3%)	
Diabetes	18 (22.8%)	20 (30.8%)	0.343
Hypertension	24 (30.4%)	28 (43.1%)	0.121
Hyperlipidemia	21 (26.6%)	23 (35.4%)	0.279
COPD	5 (6.3%)	6 (9.2%)	0.545
Prior stroke	4 (5.1%)	5 (7.7%)	0.732
Chronic kidney disease	12 (15.2%)	15 (23.1%)	0.285
Heart rate (bpm)	75.7±16.2	86.2±17.48	0.016
SBP (mmHg)	129.5±25.6	133.8±27.2	0.368
DBP (mmHg)	86.9±16.4	92.3±18.2	0.235
LVEF (%)	54.1±8.5	41.8±9.2	<0.001
LVEDD (mm)	4.7±1.1	5.0±1.2	0.211
LVEDV (mL)	104.6±23.9	108.9±26.4	0.395
HDL-C (mmol/L)	1.44±0.51	1.28±0.49	0.116
LDL-C (mmol/L)	2.75±0.93	3.13±0.99	0.089
TC (mmol/L)	3.79±1.13	4.28±1.21	0.106
TG (mmol/L)	1.42±0.57	1.81±0.56	0.337
hs-CRP (mg/L)	8.52±3.18	13.61±4.34	<0.001
BNP (pg/mL)	196.81±79.35	309.54±88.93	<0.001
CK-MB (ng/mL)	121.53±49.46	249.94±55.81	<0.001
Cystatin C (mg/L)	1.93±0.68	2.60±0.69	<0.001

The data are reported as means±SD or n (percentage). The comparisons of data between the HF and non-HF groups were done by Mann Whitney test or Fisher's exact test. COPD: chronic obstructive pulmonary disease; SBP: systolic blood pressure; DBP: diastolic blood pressure; LVEF: left ventricular ejection fraction; LVEDD: left ventricular end-diastolic diameter; LVEDV: left ventricular end-diastolic volume; HDL-C: high density lipoprotein cholesterol; LDL-C: low density lipoprotein cholesterol; TC: total cholesterol; TG: triglycerides; hs-CRP: high-sensitivity C-reactive protein; BNP: brain natriuretic peptide; CK-MB: creatine kinase-muscle/brain.

AMI patients in the HF group had significantly higher heart rate than those in the non-HF group (86.2±17.48 *vs* 75.7±16.2 bpm, P=0.016). However, blood pressure showed no significant different between groups (all P>0.05). Baseline LVEF in the HF group was lower than in the non-HF group (41.8±9.2% *vs* 54.1±8.5%, P<0.001). The LVEDD and LVEDV showed no significant difference (all P>0.05).

**Figure 2:**
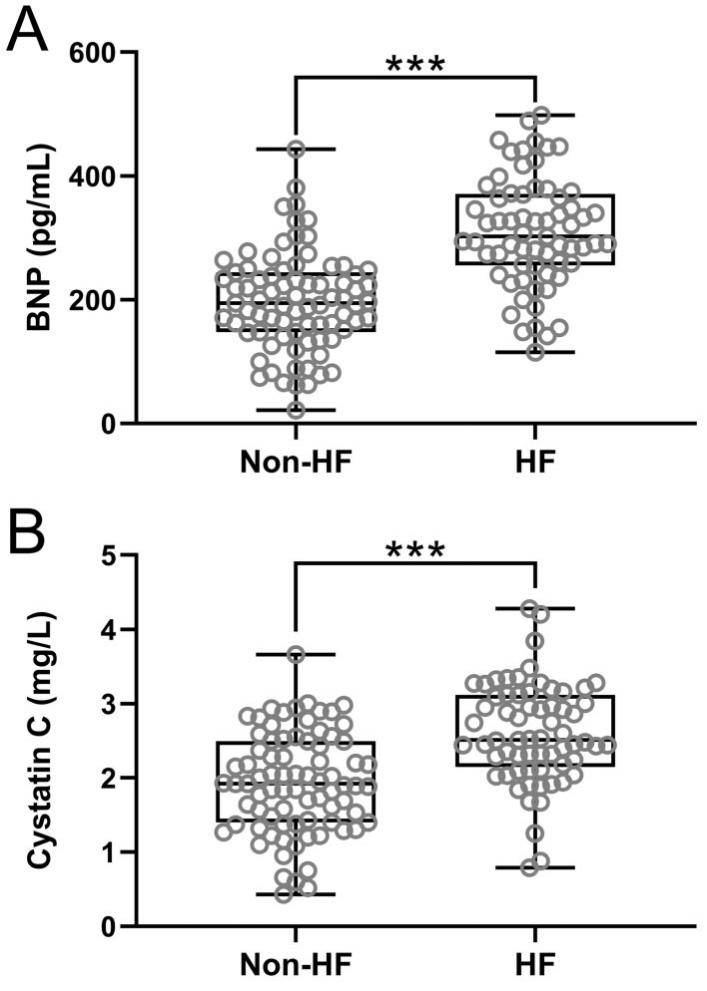
Comparisons of plasma brain natriuretic peptide (BNP) (**A**) and serum cystatin C (**B**) between patients with (n=65) and without (n=79) new heart failure (HF) onset at one-year follow up after acute myocardial infarction. Box plot was used to present the data (median and interquartile range). ***P<0.001, Mann-Whitney test.

TG, TC, LDL-C, and HDL-C had no significant difference between the two groups (all P>0.05). Patients in the HF group had higher hs-CRP levels (13.61±4.34 *vs* 8.52±3.18 mg/L, P<0.001), higher CK-MB levels (249.94±55.81 *vs* 121.53±49.46 ng/mL, P<0.001), higher BNP levels (309.54±88.93 *vs* 196.81±79.35 pg/mL, P<0.001) (Figure 2A), and higher Cys C levels (2.60±0.69 *vs* 1.93±0.68 mg/L, P<0.001) (Figure 2B).

### Multivariate logistic analysis of predictors for heart failure

The multivariate logistic regression analysis of risk factors for heart failure in AMI patients one year after undergoing emergency PCI is shown in Table 2. The results showed that high age, high heart rate, low LVEF, and high hs-CRP, BNP, CK-MB, and Cys C levels were the independent influencing factors for heart failure within one year after PCI in patients with AMI.

**Table 2:** Multivariate logistic analysis of predictors for heart failure onset at one-year follow-up after acute myocardial infarction.

	OR	95%CI	P value
High age (years)	1.246	1.101 to 1.314	0.003
High heart rate (bpm)	1.141	1.009 to 1.235	0.015
Low LVEF (%)	3.724	2.748 to 5.104	<0.001
High hs-CRP (mg/L)	1.876	1.552 to 2.095	<0.001
High CK-MB (ng/mL)	2.281	1.916 to 2.779	0.001
High BNP (pg/mL)	1.671	1.381 to 1.974	<0.001
High cystatin C (mg/L)	1.973	1.682 to 2.337	<0.001

OR: odds ratio; CI: confidence interval; LVEF: left ventricular ejection fraction; hs-CRP: high-sensitivity C-reactive protein; CK-MB: creatine kinase-muscle/brain; BNP: brain natriuretic peptide.

### Efficacy of BNP and Cys C on the prediction of heart failure in patients with AMI

**Figure 3:**
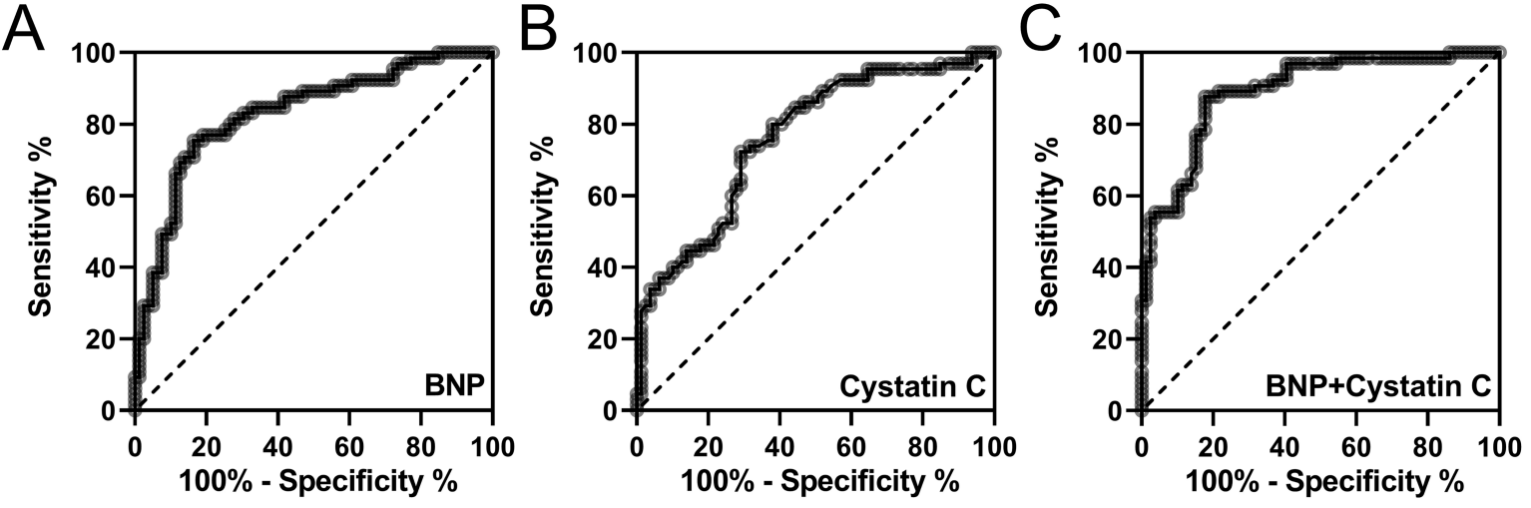
ROC analysis of plasma brain natriuretic peptide (BNP) (**A**), serum cystatin C (**B**), and combined BNP and cystatin C (**C**) for prediction of heart failure in patients after acute myocardial infarction.

The area under the curve (AUC) of BNP level in heart failure prediction was 0.829 (95%CI: 0.761 to 0.898), while the sensitivity was 75.38% and the specificity was 83.54% (Figure 3A and Table 3). The AUC of Cys C level in heart failure prediction was 0.763 (95%CI: 0.685 to 0.840), while the sensitivity was 72.31% and the specificity was 70.89% (Figure 3B and Table 3). The AUC of combined BNP and Cys C in heart failure prediction was 0.893 (95%CI: 0.842 to 0.945), while the sensitivity was 87.69% and the specificity was 82.28% (Figure 3C and Table 3).

**Table 3:** Diagnostic values in ROC analysis.

	Cut-off value	AUC (95% CI)	P	Sensitivity (%)	Specificity (%)	Youden index
BNP	256.00	0.829 (0.761 to 0.898)	<0.001	75.38	83.54	0.59
Cystatin C	2.29	0.763 (0.685 to 0.840)	<0.001	72.31	70.89	0.43
BNP+Cystatin C^+^	8.08	0.893 (0.842 to 0.945)	<0.001	87.69	82.28	0.70

CI: confidence interval; BNP: brain natriuretic peptide. ^+^Logit (BNP + cystatin C) = 0.017 * BNP + 1.739 * cystatin C.

## Discussion

The main findings of our study were: 1) Cys C and BNP levels were independent factors for heart failure within one year after PCI among AMI patients; 2) the combined BNP and Cys C were good predictors of heart failure in AMI after PCI and can be used to guide clinical decisions.

AMI is a common cardiovascular disease. The widespread use of emergency PCI has significantly reduced the mortality of AMI (9). Heart failure is a common complication of AMI, caused by heart dysfunction, and is characterized by high morbidity and mortality rates (10). Heart failure is the end stage of diverse cardiovascular diseases (11). The prevalence of heart failure in people over 65 years of age in developed countries is about 6%, and in people over 70 years of age, the prevalence is over 10% (12). Early identification of the onset of heart failure after AMI and intervention within the appropriate time window are key to improving patient prognosis. Therefore, the search for predictors of heart failure occurrence is crucial for the remission and treatment of AMI.

Patients with myocardial infarction are at higher risk of heart failure due to the progression of ventricular remodeling. The patient's heart function gradually changes from compensated to decompensated (2), which eventually leads to an enlarged left ventricle and reduced LVEF (13). Due to decreased effective arterial blood volume, pressure receptors in the aortic arch, carotid sinus, and small inlet arteries of the kidney have a deficit in cardiac output and redistribution of circulating blood volume, triggering a neuroendocrine compensatory mechanism (14). Continuous activation of the renal angiotensin system and sympathetic nervous system causes constriction of the small renal inlet arteries, which further aggravates the perfusion deficit of the kidneys and causes renal insufficiency (15).

Cys C is a small-molecule protein consisting of 120 amino acids and is an extracellular cysteine protease inhibitor that regulates the metabolism of extracellular proteins (16). Cys C levels in serum are closely related to glomerular filtration rate (GFR) (8), making Cys C an ideal GFR marker. Serum levels of Cys C contribute to the development of cardiovascular disease (17).

Cys C level was significantly higher in the HF group. Multivariate logistic analysis showed that Cys C was a predictor for heart failure in AMI patients at one-year follow up.

BNP is widely used in chronic heart failure diagnosis and treatment (18). Elevated BNP levels usually indicate heart failure and may have adverse consequences. Only a small amount of BNP exists in the blood of healthy people and is stored in the secretory granules of the atria as BNP precursor (proBNP), and BNP in the blood circulation is stable below 10 pg/mL (19).

Myocardial ischemia and necrosis after the onset of AMI lead to weakened contractility and decreased compliance, resulting in myocardial strain, accelerated heart rate, and increased atrial ventricular volume and ventricular wall tension, causing explosive synthesis and secretion of BNP (20). Therefore, the detection of BNP concentration after AMI is an important guide for clinical work. The elevation of BNP in the early stage of AMI is mainly due to the rapid cleavage of a small amount of proBNP from the atrial secretory granules and its release into the blood. In the late stage of AMI, uncoordinated myocardial contraction in the infarcted and non-infarcted regions after myocardial necrosis stimulates massive synthesis and release of BNP from the ventricles (21). Therefore, the BNP concentration increases slowly and then accelerates after AMI. In populations with declining cardiac function, ventricular compliance decreases, and basal BNP levels increase with aging (22).

There were several limitations in this research. This research was a single-center study, which might have led to selection bias. Second, this research was limited by a relatively small number of patients. Some of the patients were also lost to follow-up.

### Conclusion

We concluded that Cys C and BNP levels are independent influencing factors for heart failure after AMI. The combined BNP and Cys C might help in the prediction of heart failure in AMI patients after PCI and could be used to guide clinical decisions.

## References

[1] Gulati R, Behfar A, Narula J, Kanwar A, Lerman A, Cooper L (2020). Acute myocardial infarction in young individuals. Mayo Clin Proc.

[2] Boateng S, Sanborn T (2013). Acute myocardial infarction. Dis Mon.

[3] Saito Y, Kobayashi Y (2019). Percutaneous coronary intervention strategies in patients with acute myocardial infarction and multivessel disease: completeness, timing, lesion assessment, and patient status. J Cardiol.

[4] Bahit MC, Kochar A, Granger CB (2018). Post-myocardial infarction heart failure. JACC Heart Fail.

[5] Li N, Wang JA (2005). Brain natriuretic peptide and optimal management of heart failure. J Zhejiang Univ Sci B.

[6] de Denus S, Pharand C, Williamson DR (2004). Brain natriuretic peptide in the management of heart failure: the versatile neurohormone. Chest.

[7] Llauger L, Jacob J, Miro Ò (2018). Renal function and acute heart failure outcome. Med Clin (Barc).

[8] Ferguson TW, Komenda P, Tangri N (2015). Cystatin C as a biomarker for estimating glomerular filtration rate. Curr Opin Nephrol Hypertens.

[9] Lu Y, Yan Y, Liu X (2021). Effects of alprostadil combined with tanshinone IIa injection on microcirculation disorder, outcomes, and cardiac function in AMI patients after PCI. Ann Palliat Med.

[10] Wang D, Lv L, Xu Y, Jiang K, Chen F, Qian J (2021). Cardioprotection of Panax Notoginseng saponins against acute myocardial infarction and heart failure through inducing autophagy. Biomed Pharmacother.

[11] Abdel-Moneim A, Gaber AM, Gouda S, Osama A, Othman SI, Allam G (2020). Relationship of thyroid dysfunction with cardiovascular diseases: updated review on heart failure progression. Hormones (Athens).

[12] Chaudhry SP, Stewart GC (2016). Advanced heart failure: prevalence, natural history, and prognosis. Heart Fail Clin.

[13] Lu L, Liu M, Sun R, Zheng Y, Zhang P (2015). Myocardial Infarction: symptoms and treatments. Cell Biochem Biophys.

[14] Parmley WW (1995). Neuroendocrine changes in heart failure and their clinical relevance. Clin Cardiol.

[15] Chuang AM, Nguyen MT, Kung WM, Lehman S, Chew DP (2020). High-sensitivity troponin in chronic kidney disease: considerations in myocardial infarction and beyond. Rev Cardiovasc Med.

[16] van der Laan SW, Fall T, Soumare A, Teumer A, Sedaghat S, Baumert J (2016). Cystatin C and cardiovascular disease: a mendelian randomization study. J Am Coll Cardiol.

[17] Angelidis C, Deftereos S, Giannopoulos G, Anatoliotakis N, Bouras G, Hatzis G (2013). Cystatin C: an emerging biomarker in cardiovascular disease. Curr Top Med Chem.

[18] Gaggin HK, Januzzi JL (2013). Biomarkers and diagnostics in heart failure. Biochim Biophys Acta.

[19] Farnsworth CW, Bailey AL, Jaffe AS, Scott MG (2018). Diagnostic concordance between NT-proBNP and BNP for suspected heart failure. Clin Biochem.

[20] Cho J, Park IB, Lee K, Ahn TH, Park WB, Kim JH (2018). Statin has more protective effects in AMI patients with higher plasma BNP or NT-proBNP level, but not with lower left ventricular ejection fraction. J Cardiol.

[21] Liu Z, Ma C, Gu J, Yu M (2019). Potential biomarkers of acute myocardial infarction based on weighted gene co-expression network analysis. Biomed Eng Online.

[22] Burnett JC, Ma X, McKie PM (2019). Myocardial aging, the cardiac atria, and BNP: what does it all mean?. J Am Coll Cardiol.

